# Elbow Dislocations in the National Football League: Epidemiology and Management

**DOI:** 10.7759/cureus.19241

**Published:** 2021-11-03

**Authors:** Daniel Gibbs, Shawn Sahota, Ognjen Stevanovic, Kristina Franke, Christina Mack, Gordon Nuber

**Affiliations:** 1 Department of Orthopaedics, Heiden Orthopedics, Park City, USA; 2 Orthopaedic Surgery, Northwestern University Feinberg School of Medicine, Chicago, USA; 3 Real World Insights, QuintilesIMS, Research Triangle Park, USA

**Keywords:** epidemiology, elbow, dislocation, nfl, football (american)

## Abstract

Background

Currently, it is not known how the combined osseous and ligamentous injury of a traumatic elbow dislocation in a National Football League (NFL) athlete affects management and return to play. In this study, we aimed to describe the epidemiology, management, and return to play for elbow dislocations in NFL athletes.

Methodology

This is a descriptive observational study. A retrospective review of all elbow dislocations between 2000 and 2014 (15 seasons) was performed using the NFL Injury Surveillance System (NFLISS).

Results

Over 15 NFL seasons, 82 elbow dislocations were recorded in the NFLISS. Among players who reported surgery (n = 5), players missed an average of 73.8 days of play. Among those who did not report surgery, players missed an average of 36.1 days. The overall incidence was 0.26 dislocation events per 10,000 athlete exposures. The majority of these injuries occurred during regular-season games, in defensive linebackers and linemen, during tackling contact with another player, and most commonly on a running play.

Conclusions

This study demonstrates that an elbow dislocation is not a career-ending or season-ending injury in an NFL cohort. Information regarding incidence, positions affected, whether surgical management is utilized, and return to play will help players who sustain and physicians who treat these injuries in elite football athletes understand the impact of their injuries.

## Introduction

In active National Football League (NFL) athletes, elbow dislocations account for 5% of all upper extremity injuries and 17.6% of all elbow injuries [1]. While there have been prior investigations regarding elbow dislocations involving higher-level athletes, such as collegiate [2,3] and high school players [4], aside from one smaller study [5], detailed information about the incidence, positions affected, and events surrounding an elbow dislocation is limited in this study population. Furthermore, very little is known regarding the characteristics and management of elbow dislocations in any professional athletic cohort [6].

The elbow is a complex, constrained ginglymoid and trochoid joint; meaning it has both hinged and rotary motion. The primary stabilizers of the elbow are the ulnotrochlear joint, the anterior bundle of the medial collateral ligament (MCL), and the lateral collateral ligament (LCL). The main secondary stabilizers include the radiocapitellar joint, the joint capsule, and the extensor and flexor musculotendinous masses [7].

Elbow dislocations are characterized as simple when they occur with no associated osseous fracture, or complex when a fracture accompanies disruption of the ulnohumeral articulation. Associated fractures include fractures of the radial head and neck, coronoid, and medial and lateral epicondyles [8]. When the elbow dislocates, the injury typically occurs to all the capsuloligamentous stabilizers around the elbow [9,10]. The highly constrained nature of the elbow joint and the compressive forces generated by the muscles and tendons that cross the joint confer stability after reduction despite a significant injury to the medial and lateral ligamentous stabilizers [11]. This anatomic feature helps explain why in an analysis of 4,878 simple elbow dislocations, only 2.3% required stabilization surgery [12].

Despite previous reporting on the incidence of elbow dislocations, it is unclear how this combined osseous and ligamentous injury affects management and return to play in NFL athletes. The purpose of our study is to determine the incidence of elbow dislocations among NFL athletes; to determine the rates of surgical and nonsurgical management of these injuries; and to compare the time missed for injuries treated nonoperatively versus the time missed for injuries requiring surgical intervention.

## Materials and methods

After approval by the NFL Injury and Safety Panel and our Institutional Review Board, a retrospective review of all elbow dislocations between 2000 and 2014 (15 seasons) was performed using the NFL Injury Surveillance System (NFLISS). This database includes information on injury type and severity, the onset of injury, player return to participation status due to days missed and surgery, position and activity during which the injury occurred, and other factors related to playing conditions. Within the timeframe of this study, injuries were considered “reportable” to the surveillance system if: (1) the injury resulted in time lost from game or practice including being removed from the remainder of the session during which the injury was sustained, or (2) the injury was classified as reportable regardless of time loss (concussion, fracture, dental injury, heat-related illness, injury required medical intervention or special equipment) [13].

Athletes can experience more than one injury at a time, and in such a case, athletic trainers designate a single injury as “primary” for the NFLISS. To provide a comprehensive overview of elbow dislocation injuries to NFL players, this study presents the overall counts of elbow dislocations and considers all injuries regardless of whether they were reported as primary, secondary, tertiary, or quaternary. The exception to this is for return to play statistics in which only elbow dislocations counted as the primary injury were included to limit as much as possible confounding factors on return to play statistics.

In this study, elbow dislocations were defined as elbow dislocations or elbow fracture-dislocations that occurred during preseason, regular-season, and postseason practices and games. Incidence of elbow dislocation injuries per 10,000 athlete exposures was evaluated. An athlete exposure is defined as a single NFL athlete participating in a single practice or game. Athlete exposures were calculated based on possible participation for any length of time in a practice or game. We assumed 53 players per team and 105 practices per year. We used actual numbers of games and teams per year for all seasons (preseason, regular season, postseason).

We evaluated each injury by player position, setting (game vs. practice), type of play (passing, running, fumble, kick-off return, punt return, field goal attempt, extra point attempt, unknown), management (operative vs. nonoperative), and total days missed due to the injury.

## Results

There were 82 elbow dislocations recorded in the NFLISS over 15 NFL seasons (2000-2014). Of these, 69 (84.1%) injuries occurred during a game and 13 (15.9%) occurred during practice. In total, 21 (25.6% of all injuries) dislocations occurred during the preseason, 60 (73.2%) occurred during the regular season, and 1 (1.2%) occurred in the postseason. Of the 21 preseason dislocations, 11 (52.4%) occurred in practice. Of the 60 regular-season dislocations, by contrast, 2 (3.3%) occurred in practice (Table 1). The left elbow was involved in 51 (62.2%) athlete injuries and the right elbow in 31 (37.8%) athlete injuries.

**Table 1:** Demographics of elbow dislocations in active National Football League athletes by season and session (2000-2014).

Season	Session	Total elbow dislocations (n = 82)
Preseason	Practice	11 (13.4%)
	Game	10 (12.2%)
Regular season	Practice	2 (2.4%)
	Game	58 (70.7%)
Postseason	Practice	0 (0%)
	Game	1 (1.2%)
Total	Practice	13 (15.9%)
	Game	69 (84.1%)

Focusing specifically on the 69 athletes injured during a game, 13 (18.8%) played on offense, 45 (65.2%) played on defense, and 11 (15.9%) played on special teams at the time of the injury (Table 2). Overall, 60 (87.0%) dislocations were the result of contact with another player; 7 (10.1%) dislocations were the result of contact with the player surface, equipment, nonplayer personnel, or contact unknown; and 2 (2.9%) were the result of noncontact injuries (Table 3). In total, 16 (23.2%) dislocations occurred on a pass play, 37 (53.6%) occurred on a run play, 1 (1.4%) occurred on a fumble play, 6 (8.7%) occurred on a kick return, and 4 (5.8%) occurred on a punt return. The game activity was not known in 5 (7.2%) cases (Table 4).

**Table 2:** Demographics of elbow dislocations in active National Football League athletes by position (2000-2014).

Position		In-game elbow dislocations (n = 69)
Offense		
	Offensive line	2 (2.9%)
	Running back	2 (2.9%)
	Tight end	4 (5.8%)
	Quarterback	1 (1.4)
	Wide receiver	4 (5.8%)
Defense		
	Secondary	10 (14.5%)
	Defensive line	16 (23.2%)
	Linebacker	19 (27.5%)
Special teams		11 (15.9%)

**Table 3:** Demographics of elbow dislocations in active National Football League athletes by contact type (2000-2014).

Contact type		In-game elbow dislocations (n = 69)
Contact with another player		
	Tackling	38 (55.1%)
	Tackled	7 (10.1%)
	Blocking	3 (4.3%)
	Blocked	11 (15.9)
	Unknown	1 (1.4%)
Contact with playing surface		7 (10.1%)
Noncontact		2 (2.9%)

**Table 4:** Demographics of elbow dislocations in active National Football League athletes by activity (2000-2014).

Activity		In-game elbow dislocations (n = 69)
Pass play		16 (23.2%)
Run play		37 (53.6)
Fumble		1 (1.4%)
Kick-off return		6 (8.7%)
Punt return		4 (5.8%)
Unknown		5 (7.2%)

In-game elbow dislocations over the 15-year period evaluated revealed an incidence of 1.31 per 10,000 athlete exposures. Athletes were less likely to dislocate their elbows during practice with an incidence of 0.05 per 10,000 athlete exposures. Overall incidence was 0.26 dislocation events per 10,000 athlete exposures.

Only five elbow dislocations required surgical management. None of these five injuries were reported as complex dislocations (dislocations associated with a fracture). Those requiring surgery had a mean time missed of 73.8 days (range = 3-150, SD = 61.9). Those dislocations that did not report surgery had a mean time missed of 36.1 days (range = 0-163, SD = 33.4) (Table 5). The total return to play for all athletes was 38.5 days (SD = 36.4) Time missed was defined as the number of days it took for the athlete to return to full participation in football activities from the injury.

**Table 5:** Return to play statistics by reported surgery after elbow dislocation in National Football League athletes (2000-2014). ^1^Only elbow dislocations reported as the primary injury (if multiple injuries occurred) are included in time lost calculations (n = 81, 98.8%). ^2^Two elbow dislocations are excluded from analysis due to missing return to participation dates. Surgeries that occurred outside of National Football League medical staff treatment may not have been reported, particularly in the earlier years of the study.

Return to play^1^	n	Days missed	Days missed	Range
		Mean (SD)	Median	
Reported surgery	5	73.8 (61.9)	64	3–150
Surgery not reported^2^	74	36.1 (33.4)	25	0–163
Total	79	38.5 (36.4)	28	0–163

## Discussion

Clinically, an elbow dislocation causes substantial trauma to the musculoligamentous complex surrounding the elbow [9,10]. Considering the substantial soft tissue injury, it is surprising that, in the general population, only 2.3% of simple dislocators required stabilization surgery [12]. Our study demonstrates a high return to play, a low rate of surgical management consummate with that of the general population, and 36.1 days of missed play for nonoperatively treated athletes, despite the significant trauma sustained by the elbow.

In the general population, the elbow is the second most commonly dislocated major joint after the shoulder [14]. Stoneback et al. evaluated a random sample of all patients presenting to 102 emergency departments using the National Electronic Injury Surveillance System and found an incidence of 5.21 elbow dislocations per 100,000 person-years. They found that 44.5% of all elbow dislocations in patients older than 10 years occurred as a result of sports. Moreover, they found that nearly 22% of all elbow dislocations occurred in young male football players [15].

Although there have been select studies investigating elbow dislocations in higher-level athletes, namely, at high school [4] and college level [2,3], there is a paucity of literature addressing this type of injury in professional football athletes. Carlisle et al. reported on NFL athletes and upper extremity injuries in general [1]. They reported a mean number of days lost of 14 for elbow ligamentous and joint instability (which included sprains, dislocations, and subluxations). An elbow sprain or subluxation has a different post-injury recovery than an elbow dislocation. Therefore, our mean days missed of 36.1 in patients treated nonoperatively is a more reliable indicator of time missed for elbow dislocations. Chang et al. performed a similar study to ours, where they also reviewed the NFLISS database on elbow dislocations, albeit in a shorter time frame, and, therefore, resulting in a smaller cohort [5]. While the incidence remained similar, their mean time lost appeared to be shorter than found in our study, whether the patient underwent surgery (46.5 days) or if the conservative management was pursued (25.1 days).

Little data exist to guide team physicians in post-injury treatment for athletes with simple elbow dislocations. Iordens et al. performed a multicenter randomized clinical trial comparing early mobilization versus plaster immobilization for simple elbow dislocations [16]. Comparing mobilization at two days post-injury to three weeks of plaster immobilization, the authors found improved patient-reported and functional outcome scores, as well as a better total arc of motion at six weeks for those treated with early mobilization. Protzman evaluated 49 simple elbow dislocations in active US Military Academy cadets [17]. He found that those immobilized longer than five days had worse motion and worse disability compared to those immobilized for less than five days. No high-level evidence exists for the treatment of simple elbow dislocations in professional contact athletes. Our data show that the average return to play for simple elbow dislocations that do not require surgery is approximately weeks. High-level research should be performed to evaluate which treatment modalities are best for returning athletes to play the fastest with the lowest morbidity.

This study provides information regarding elbow dislocations in a professional football cohort. This study certainly has limitations. Because it is a database review, specifics about both the injury and the treatment are limited. Data were available for whether the patient had surgical treatment of the injury; however, specific details on the type of surgery or surgical findings were not documented. Surgeries are reported by the medical staff of the Club, and, in this study, if surgery was not reported, we assumed it did not occur. However, as in the 15-year period of this study, there is known under-ascertainment of surgery due to players receiving surgery outside of the Club staff and often during the offseason. Specific information about treatment measures other than surgery was not recorded in the NFLISS during the study duration. Finally, numerous factors play a role in return to play, including the team physician’s guidance, rehabilitation protocols, and the presence of ongoing symptoms. We could not fully evaluate these factors as they related to return to play in this cohort.

More work is needed to characterize the long-term consequences of an elbow dislocation in this athletic population; however, understanding the incidence, treatment, and return to play will help players who sustain and physicians who treat these injuries in high-level football athletes understand the significance of their injuries. Future directions for this research topic include examining specific nonoperative treatments to evaluate which worked best in returning the athlete to play as well as evaluating the risk factors for prolonged return to play.

## Conclusions

Elbow dislocation is a rare event among professional football players. Nonetheless, this ligamentous and osseous injury imparted to the elbow can be substantial. This study demonstrates that an elbow dislocation is not a career-ending or season-ending injury in an NFL cohort. Most of the dislocations occur among players playing on defense, namely, linebackers and defensive linemen. The average return to play, regardless of whether surgery is needed, is just over five weeks with a median return to play of four weeks. Furthermore, in the vast majority of injuries, despite significant trauma sustained by the elbow, nonoperative management was pursued, which resulted in a successful return to play after five weeks. These statistics are useful to physicians as they may guide management and assist with counseling athletes who sustain these injuries.
